# Ketamine study: Protocol for naturalistic prospective multicenter study on subcutaneous ketamine infusion in depressed patients with active suicidal ideation

**DOI:** 10.3389/fpsyt.2023.1147298

**Published:** 2023-03-09

**Authors:** Ana Paula Anzolin, Jeferson Ferraz Goularte, Jairo Vinícius Pinto, Paulo Belmonte-de-Abreu, Luciane Nascimento Cruz, Victor Hugo Schaly Cordova, Lucas Sueti Magalhaes, Adriane R. Rosa, Keila Maria Cereser, Márcia Kauer-Sant’Anna

**Affiliations:** ^1^Graduate Program in Biological Sciences, Biochemistry, Universidade Federal do Rio Grande do Sul (UFRGS), Porto Alegre, Rio Grande do Sul, Brazil; ^2^Laboratory of Molecular Psychiatry, Hospital de Clínicas de Porto Alegre (HCPA), Porto Alegre, Rio Grande do Sul, Brazil; ^3^Graduate Program in Psychiatry and Behavioral Sciences, Universidade Federal do Rio Grande do Sul (UFRGS), Porto Alegre, Brazil; ^4^University Hospital, Universidade Federal de Santa Catarina (HU-UFSC), Florianópolis, Santa Catarina, Brazil; ^5^Department of Psychiatry, Universidade Federal do Rio Grande do Sul (UFRGS), Porto Alegre, Rio Grande do Sul, Brazil; ^6^Psychiatry Service, Hospital Moinhos de Vento, Porto Alegre, Rio Grande do Sul, Brazil; ^7^School of Pharmacy, Universidade Federal do Rio Grande do Sul (UFRGS), Porto Alegre, Rio Grande do Sul, Brazil; ^8^Department of Pharmacology, Universidade Federal do Rio Grande do Sul (UFRGS), Porto Alegre, Rio Grande do Sul, Brazil; ^9^CNPq, FAPESP, CAPES, National Institute for Science and Technology in Translational Medicine (INCT-TM), São Paulo, Brazil

**Keywords:** suicide, bipolar depression, unipolar depression, depression, ketamine

## Abstract

**Background:**

Psychiatric disorders are associated with more than 90% of reported suicide attempts worldwide, but few treatments have demonstrated a direct effect in reducing suicide risk. Ketamine, originally an anesthetic, has been shown anti-suicide effects in clinical trials designed to treat depression. However, changes at the biochemical level were assessed only in protocols of ketamine with very limited sample sizes, particularly when the subcutaneous route was considered. In addition, the inflammatory changes associated with ketamine effects and their correlation with response to treatment, dose-effect, and suicide risk warrant further investigation. Therefore, we aimed to assess whether ketamine results in better control of suicidal ideation and/or behavior in patients with depressive episodes and whether ketamine affects psychopathology and inflammatory biomarkers.

**Materials and methods:**

We report here the design of a naturalistic prospective multicenter study protocol of ketamine in depressive episodes carried out at *Hospital de Clínicas de Porto Alegre* (HCPA) and *Hospital Moinhos de Vento* (HMV). The study was planned to recruit adult patients with Major depressive disorder (MDD) or Bipolar disorder (BD) types 1 or 2, who are currently in a depressive episode and show symptoms of suicidal ideation and/or behavior according to the Columbia-Suicide Severity Rating Scale (C-SSRS) and have been prescribed ketamine by their assistant psychiatrist. Patients receive ketamine subcutaneously (SC) twice a week for 1 month, but the frequency can be changed or the dose decreased according to the assistant physician’s decision. After the last ketamine session, patients are followed-up *via* telephone once a month for up to 6 months. The data will be analyzed using repeated measures statistics to evaluate the reduction in suicide risk as a primary outcome, as per C-SSRS.

**Discussion:**

We discuss the need for studies with longer follow-ups designed to measure a direct impact on suicide risk and that additional information about the safety and tolerability of ketamine in particular subset of patients such as those with depression and ideation suicide. In line, the mechanism behind the immunomodulatory effects of ketamine is still poorly understood.

**Trial registration:**

https://clinicaltrials.gov/, identifier NCT05249309.

## Introduction

Depression is a chronic, recurrent, and highly prevalent condition associated with functional disability and compromised physical health (1). Its etioloy is multifactorial and combines endogenous susceptibility with exposure to environmental stressors (1, 2). The association between depression and suicidal behavior has been widely described in literature; for example, according to the WHO (3), psychiatric illnesses are associated with more than 90% of suicide ideation cases and are responsible for 90% of deaths by suicide reported cases worldwide (3, 4). Furthermore, studies across different populations have confirmed the relationship between depression and suicide (5–7): population-based investigations in the United States (5, 8), Canada (6), and China (7) indicate that depression is the main nosological entity associated with suicidal ideation, suicidal plans, and suicide attempts. Despite these facts, treatments for depression that also impact suicidal behavior are scarce; thus, drugs with anti-suicide effects are highly desirable and one of the main research needs in psychiatry (4).

There is an increasing interest in the benefits of ketamine, its racemic compound, and its enantiomers [i.e., S-ketamine (esketamine) (9) and R-ketamine (arketamine) (10) for the treatment of psychiatric disorders. Indeed, intranasal esketamine for treating depression was recently approved by the Food and Drug Administration (FDA) and European Regulatory Authorities (11, 12) for treating Major depressive disorder (MDD). Ketamine acts on glutamate, the principal excitatory neurotransmitter, as a non-competitive antagonist on the *N*-methyl-*D*-aspartate (NMDA) receptor; the effectiveness of glutamate modulating agents in the treatment of mood disorders regulates of glutamatergic neurotransmission, contributing to the pathophysiology of depression, as well as to the mechanisms of antidepressants (13).

Glutamate acts pre- and post-synaptically through the activation of several receptors. Ionotropic glutamate receptors-NMDA, alpha-amino-3-hydroxy-5-methyl-4-isoxazolepropionic acid (AMPA) and kainate (KA)–are channels that allow the influx of ions into the cell, regulating polarization of the neuronal surface, which activates intracellular signaling cascades. It is believed that NMDA and AMPA are directly involved in the antidepressant actions of ketamine (14). The pharmacokinetic characteristics of ketamine allow its administration by various routes, including intravenous (IV) (15, 16), subcutaneously (SC) (17, 18), intranasal (11, 12), oral (19, 20), sublingual (21), and intramuscular (22). The SC route of administration has comparable efficacy to conventional IV infusion but fewer side effects (23, 24). In a recent systematic review (18) that included 12 studies (two randomized clinical trials, five case reports and five retrospective studies), the authors observed that racemic ketamine and its enantiomer esketamine, *via* SC, seems to be a promising treatment in depression, given its efficacy and tolerability. The literature has already verified that doses of 0.5 mg/kg are unsuitable for patients with chronic depression (25). Repeated and staggered doses of ketamine (0.5 mg/kg for the first three infusions to 0.75 mg/kg for the last infusions) reinforced the antidepressant and antisuicidal properties of ketamine in a sample of patients with severe depression (25–27).

Some studies have associated proinflammatory cytokines with the severity of depressive symptoms (28, 29). Innate immune cells present in the central nervous system (CNS), such as microglia, participate in the process of neuroinflammation; when this process is activated, the production of cytokines that affect synaptic plasticity in regions important for mood regulation increases (19). Based on pharmacological properties and animal studies (30), it is hypothesized that depressed patients with higher levels of inflammation is more responsive to ketamine treatment (31). Furthermore, in an animal model of treatment-refractory depression with chronic administration of adrenocorticotropic hormone (ACTH), animals that responded to ketamine exhibited higher baseline plasma concentrations of C-reactive protein (CRP) and tumoral necrosis factor-α (TNF-α) (32, 33). Blood biomarkers [TNF-α, Interleukin (IL)-6] predicted a favorable antidepressant response to Ketamine administration in a small sample of depressed patients. Other studies report that increased body mass index (BMI) and plasma concentrations of adipokine (both associated with inflammation) correlated with the ketamine response, in other words, lower baseline adiponectin levels correlated with superior antidepressant response to ketamine (percent change from baseline) at 230 min post-infusion [Montgomery-Åsberg Depression Rating Scale (MADRS): *r* = 0.25, *p* = 0.03; Hamilton Depression Rating Scale (HAM-D): *r* = 0.22, *p* = 0.051] and at day 1 (MADRS: *r* = 0.28, *p* = 0.01; HAM-D: *r* = 0.34, *p* = 0.002) (13, 34).

Patients with depression are also at increased risk for developing metabolic and cardiovascular diseases, being, on average, 1.58 times more likely to have metabolic syndrome (MS) compared to the general population (35). Among the hormones secreted by adipose tissue, leptin seems to be involved with depressive disorders (36–38). In addition, some works have investigated the involvement of central and peripheral leptin as a potential biomarker for suicide risk (39).

New biomarkers are also essential to predict the outcome of treatment in the future with the application of conventional antidepressants and anti-inflammatory drugs. For example, alterations in the expression of sirtuin 3 (SIRT3) are associated with the pathophysiology of depressive disorders (40). Similarly, serum levels of soluble urokinase plasminogen activator receptor (suPAR) are positively correlated with inflammatory proteins previous reported in mood disorders, such as tumor necrosis factor-alpha [TNF-α_ and ultra-sensitive C-reactive protein (us-CRP) (41)]. Moreover, studies have observed that high levels of suPAR were associated with a higher probability of depression diagnosis and recent suicide attempts (42, 43).

Therefore, given the potential challenges of conducting a definitive randomized control trial (RCT) of ketamine as a rapid-onset antidepressant in suicidal ideation, a feasibility study is needed to inform tolerability, acceptability, safety, effect, as well as the better understanding of the biochemical changes of ketamine in the treatment of suicide risk in patients with depression. In addition, these data could serve as the basis for the larger RCT using an individualized dose ketamine approach.

### Aims

This article aims to describe the protocol of a multicenter prospective naturalistic study, which allows an analysis of the response to ketamine *via* SC in relation to the treatment of suicidal ideation and behavior. We hypothesize that ketamine, through its mechanisms of action on NMDA and neuroplasticity, would reduce suicidal ideation or/and behavior in patients with a depressive episode, according to the Columbia Suicide Severity Rating Scale (C-SSRS) and other rating scales.

### Objectives

Our main objective is to evaluate whether ketamine can reduce the frequency and intensity of suicidal ideation or behavior and improve depressive symptoms in patients with depressive episodes. Our secondary aims are the investigation of the impact of ketamine on other psychoathology symptoms, clinical factors, inflammatory biomarkers, and metabolic factors.

## Materials and methods

### Study design and setting

This is an observational naturalistic, prospective multicenter study performed in two reference centers in ketamine treatment in Porto Alegre, Brazil. The participants were recruited in our reference due to their depression and active suicide ideation or behavior. The ongoing ketamine study started data collection on July 2021 with a target of 45 participants. Data include clinical and psychiatric assessment, blood sampling, and diet on site with a follow-up assessment at 6 months performed by phone call. In addition, an exploratory analysis assesses the risk of suicide throughout ketamine treatment based on subgroups of interest.

### Sample size calculation

The sample size of 45 participants over 2 years is projected to be an appropriate number to inform study feasibility; the sample size calculation was performed using the WINPEPI program, version 11.65 to detect a difference of 1 point on the C-SSRS scale, considering results from previous studies (44) with a power of 80% at a significance level of 0.05.

### Population and eligibility criteria

The target population are adult patients diagnosed with Major depressive disorder (MDD) currently in depressive episodes, were diagnosed using the Diagnostic and Statistical Manual of Mental Disorders–fifth edition, DSM-5 (45), and confirmed using the Mini International Neuropsychiatric Interview (MINI; updated Version 7 for DSM-5) (46).

All patients are treated with subcutaneous (SC) ketamine and continue to use psychiatric medications prescribed by their attending physician. Information on medications (single or in combination) as well as dosage were collected using a structured questionnaire (Appendix 1). Patients did not receive psychotherapy and/or physiotherapy.

The inclusion criteria are (1) adult patients (≥ 18 years); (2) that meet the DSM-5 diagnostic criteria for MDD, BD-1, or BD-2 currently in a depressive episode; (3) with a total score on the Montgomery-Åsberg Depression Rating Scale (MADRS) ≥ 12 and score on items 1 (apparent sadness) and 2 (expressed sadness) ≥ 2 during the triage period (baseline); (4) and a total Young Mania Rating Scale (YMRS) score ≤ 11 during baseline; (5) having current symptoms of suicidal ideation or suicidal behavior, according to the Columbia Suicide Severity Rating Scale (C-SSRS) score ≥ 1; (6) indication/prescription of their assistant physician for the use of SC ketamine; (7) use effective contraceptive methods for heterosexual women of childbearing age; (8) patients with BD-1 is taking lithium, valproic acid, or an atypical antipsychotic at therapeutic doses for at least 4 weeks before the initial assessment; (9) patients with BD – 2 is taking lithium, valproic acid, lamotrigine, or an atypical antipsychotic at therapeutic doses for at least 4 weeks before the initial assessment; (10) can provide consent and comply with study procedures.

The exclusion criteria are (1) Patients with an unstable, defined, or suspected systemic medical condition; (2) Women who are pregnant, breastfeeding or planning to become pregnant within the next year; (3) Patients who do not tolerate the use of ketamine or with previous side effects associated with medications; (4) Inability to comply with informed consent or treatment protocol needs; (5) Patients with psychotic symptoms (according to DSM-5 criteria); (6) Patients with a current diagnosis of any substance use disorder according to the DSM-5 Criteria, except for smoking; (7) Patients with immune, inflammatory, cancer or infections.

Withdrawal Criteria: (1)Patients not using the medication or being considered non-adherent by the responsible clinician; (2) Patients who stop taking contraceptives or become pregnant; (3) In case of modifying doses or adding/deleting medication, patients be kept in the study, but the changes be counted as a primary endpoint; (4) Serious adverse reactions; (5) Withdrawal of consent by the patient; (6) Patients with manic or psychotic episodes as clinically assessed and according to DSM-5 criteria.

### Comparator

The recruitment of the control group occurs in the form of an invitation to blood donors at the HCPA Blood Bank and blood collection performed in the routine collection performed during the blood donation. This group included only in biochemical analyzes to compare the levels of interleukin 6 (IL-6), IL-10, IL-1β, TNFα, SIRT3, suPAR, us-CRP, and leptin of depressed patients with levels of healthy subjects.

The control group consists of 45 healthy volunteers (age ≥ 18). The inclusion criteria for the healthy controls are: (1) Not having a history of psychiatric or neurological diseases; (2) Do not present unstable clinical illnesses or autoimmune diseases; (3) Not being pregnant or breastfeeding.

### Interventions

**FIGURE 1:**
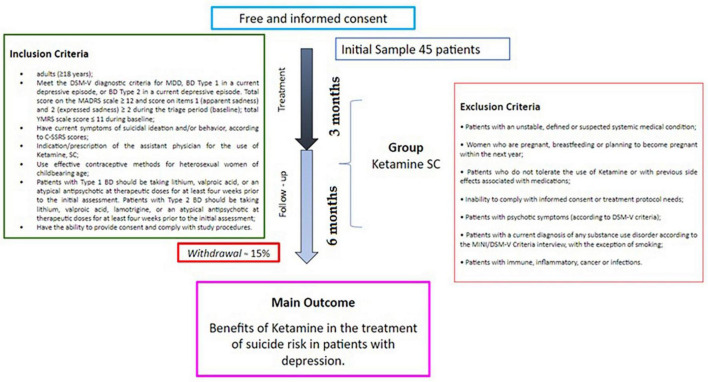
Study protocol. The protocol may have its care attention time changed/decreased, due to the amounts of ketamine sessions (determined by the attending physician), after the end of these sessions the patient is monitored once a month to 6 months, *via* telephone.

**FIGURE 2:**
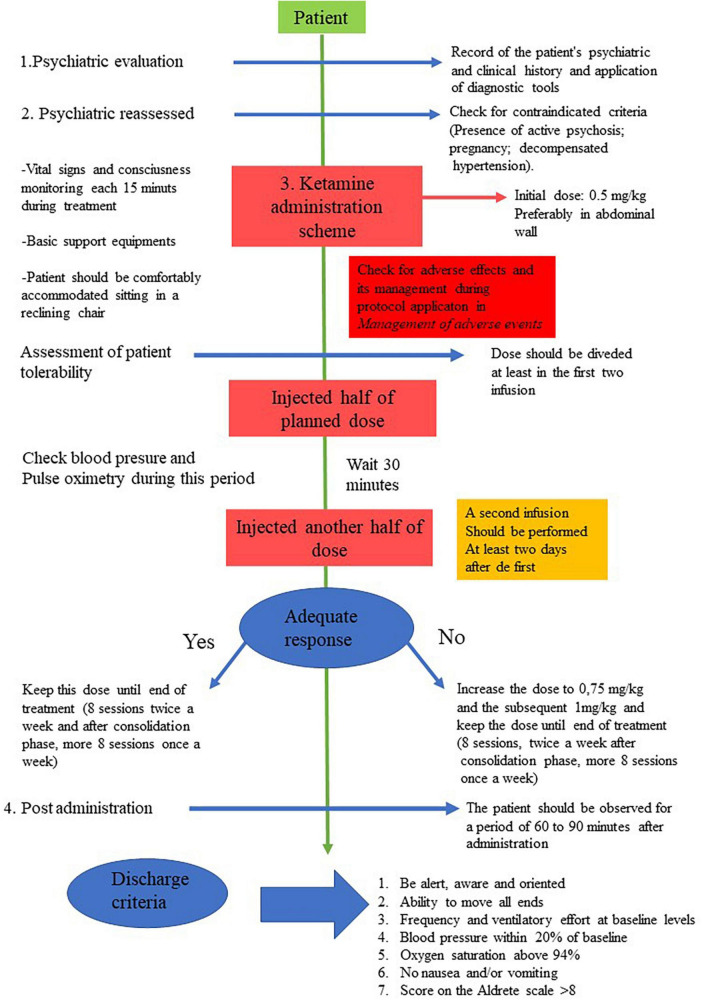
Graph scheme of ketamine protocol.

The study procedure is illustrated in Figures 1, 2. The application of ketamine for the study follows the routine related to the care in force applied to patients for treatment with ketamine at HCPA and HMV.

The psychiatrist reassesses the patient to exclude current criteria that contraindicate treatment with ketamine. In addition, it is verified whether the patient has ingested solids for at least 6 h and at least 2 h before the procedure. Finally, the patients are comfortably accommodated sitting in a reclining chair or lying on a stretcher, and vital signs are checked (blood pressure measurement, digital oximetry, and heart rate).

The medication is administered at an initial dose of 0.5 mg/kg. The nursing staff with undiluted ketamine prepare the syringe for SC administration; the psychiatrist administers the SC injection, preferably in the abdominal wall. The tolerability of the patient treated for the first time with ketamine is evaluated by dividing the dose administered at least in the first two infusions and whenever the dose is increased. In this case, the medication is injected using half of the planned dose and the other half 30 min after or when there is remission of adverse events. Blood pressure and pulse oximetry are checked during this period.

The initial SC infusion is 0.5 mg/kg ketamine; if there is no adequate response with the dose of 0.5 mg/kg, a second infusion is performed at least 2 days after the first, using 0.75 mg/kg and the subsequent 1 mg/kg. If the patient responds adequately to those doses (0.5 or 0.75 mg/kg), it is repeated throughout the course of treatment; these eight sessions, twice a week. Then, after a consolidation phase of 8 more sessions, once a week. The general and clinical data of the patients is obtained in person, along with the care procedure. After the end of the ketamine sessions, the psychiatric scales are applied *via* telephone once a month until the 6th month. In the first and last session of Ketamine (beginning and end of treatment), peripheral blood is collected from patients (15 mL) by a technician trained in blood collection.

### Blood sampling

Blood samples are collected from each patient and allowed to clot in blood collection tubes with no additive. Subsequently, whole blood is centrifuged for 10 min at 1,000 x*g* and serum is removed, aliquoted and stored at −80°C until assayed. Blood samples are collected from each patient in an anticoagulant tube. Subsequently, the blood is centrifuged for 10 min at 1,000 mg. and the plasma is removed, aliquoted and stored at −80°C until the time of the assay.

### Measurements

#### Primary outcome measures

##### C-SSRS

To measure the risk of suicide and the improvement in suicidal ideation with ketamine, we used the Brazilian version of the C-SSRS (translated and validated to Brazilina Portuguese). The C-SSRS has different versions that assess symptoms in different periods, depending on the characteristics of the study. In the present study, we use the baseline/screening version at the beginning of the first session, which assesses the worst period of suicidal ideation during life and in the last month. For later measurements (before each ketamine application), we use the modified version for use in serial assessment, which tracks symptoms since the last assessment.

The C-SSRS is applied by the researcher through a semi-structured interview and is divided into four subscales: (a) severity of suicidal ideation (5-point ordinal scale); (b) intensity of ideation (5-point ordinal scale); (c) suicidal behavior [nominal scale with binary response (yes/no)]; (d) lethality of effective attempts. The researchers performed the necessary training to apply the C-SSRS scale.

#### Secondary outcome measures

These questionnaires below are applied before all ketamine sessions and, after that, *via* telephone once a month until the 6th month. In addition, the Childhood Trauma Assessment Questionnaire (CTQ) is applied before the first ketamine session.

##### Childhood Trauma Assessment Questionnaire (CTQ)

The CTQ (47) is a self-assessment instrument for exposure to abuse situations up to fifteen years of age. It consists of 28 items, classifiable on a 5-point Likert scale, originating from the 70-item long version developed by Bernstein et al. (47). Items that describe childhood experiences are classified according to how often they occurred: 1–never, 2–a few times, 3–sometimes, 4–often or 5–always, being formulated with experiences of abuse or adequate care during childhood.

##### Young Mania Rating Scale (YMRS)

The YMRS (48), translated and adapted to Brazilian Portuguese (44), is used to evaluate the appearance of manic symptoms as an adverse effect of the use of ketamine. The YMRS is a scale the researcher applies through direct observation and unstructured interviews. This scale contains eleven items, and the score ranges from 0 to 62. A score less than or equal to 12 indicates no manic episode.

##### Montgomery-Åsberg Depression Rating Scale (MADRS)

In its final version, the MADRS Scale (49) consists of ten items that do not include somatic or psychomotor symptoms. This characteristic makes it a more suitable scale to assess patients with general medical comorbidities, as it reduces the risk that symptoms resulting from somatic illness are counted as depressive symptoms. The evaluator can score defined scale grades (0, 2, 4, 6) or intermediate categories (1, 3, 5).

##### Hamilton Depression Rating Scale (HAM-D)

To measure ketamine effectiveness on the severity of depressive symptoms, we also use the seventeen-item version of the HAM-D (49) translated and adapted to Brazilian Portuguese, with a structured interview guide. The HAM-D is a scale whose total score ranges from 0 to 52. Although the author has not proposed a standard cutoff point, in practice, scores above 24 are considered to identify a severe depressive episode; between 18 and 24, moderate depressive episode; between 7 and 17, mild depressive episode and below 7, no depressive episode or remission (50).

##### Brief psychiatric rating scale (BPRS)

This scale assesses the presence and level of severity of psychotic symptoms, emotional states, and psychomotricity disorders, among other symptoms (51). The BPRS score adopted is based on the criteria suggested by Elkis et al. (51), with scores ranging from 0 to 6 for each item. Thus, 0 (zero) means absence or non-observation of the symptom and six corresponds to the most severe level. Intermediate values correspond, in turn, to intermediate levels of severity. For the evaluation of mental alterations compatible with disorders of a psychotic nature, the total BPRS score is considered.

##### Functional assessment short test (FAST)

We use the translated and adapted version for Brazil (52). It is a hetero-applied instrument for the objective and multidimensional assessment of functionality related to the last fifteen days. It consists of 24 items, divided into six specific subscales. Autonomy refers to the subject’s ability to perform actions alone or to make their own decisions. Occupational functioning refers to the subject’s ability to maintain a regular job, to have a stable performance and to work in an area compatible with their qualification and position at work. Cognitive functioning concerns the subject’s ability to concentrate, make simple mental calculations, solve routine problems, learn new information and remember this learned information. Financial skills involve the subject’s ability to manage their finances in a balanced way; the item “interpersonal relationships” refers to the quality of relationships with friends and family, the ability to participate in social activities and sexual relationships, and the ability to defend personal ideas and opinions. Leisure activities relate to performance in physical activities (sports, exercise) and having activities. The score is determined by the sum of the items, which range from 0 (indicating no limitation) to 3 (indicating severe limitation) (53).

##### Anthropometric measurements

###### a) Body weight

Body weight is evaluated before the first application of ketamine, in the fourth, eighth and twelfth weeks after the first application of ketamine. Body weight is measured with individuals barefoot, wearing as little clothing as possible and positioned in the center of the platform during the reading. An electronic scale with a maximum capacity of 150 kg and a precision of 0.1 kg is used.

###### b) Stature

Height is evaluated before the first application of ketamine. To perform the measurement, an anthropometric ruler fixed to the wall is used. Height is considered as the distance from the sole of the bare feet to the top of the head, compressing the hair, with the patient in a vertical position, on the flat surface, looking fixed on the horizon (54).

###### c) Body mass index (BMI)

Body mass index (BMI) is calculated from weight (kg) and height (m) data using the following formula:


$$BMI=Weight\;(Kg)÷Height\;{\left(m\right)}^{2}$$

For the classification of BMI, the cutoff points established by the WHO is used, where: BMI < 18.5 Kg/m^2^ is classified as low weight; BMI 18.5–24.9 Kg/m^2^ is classified as adequate weight; BMI 25–29.9 Kg/m^2^ is classified as overweight; BMI 30–34.9 Kg/m^2^ is classified as class I obesity; BMI 35–39.9 Kg/m^2^ is classified as class II obesity; BMI > 40 Kg/m^2^ is classified as class III obesity.

###### d) Waist circumference (WC)

Waist circumference is evaluated before the first application of ketamine, in the fourth, eighth and twelfth weeks after the first application of ketamine. WC is measured with the aid of an inelastic measuring tape 1.5 m long and accurate to 0.1 cm. The measurement is performed with the patient standing in an upright position, abdomen relaxed, arms extended along the body and feet separated at 25–30 cm. The midpoint between the iliac crest and the lower edge of the last rib, in an orthostatic position, without clothes on the chest and at the end of expiration (54). The reference value used is the one proposed by the IDF (55).

###### e) Blood pressure

Blood pressure is measured before the first application of ketamine, in the fourth, eighth and twelfth weeks after the first application of ketamine. The measurement is performed according to the HCPA and HMV nursing protocol.

###### f) Inflammatory profile of the diet

The Dietary Inflammatory Index (DII) is calculated according to previous studies (56, 57) and from the average of food recalls of the last 24 h (R24h) to the interview with intervals between them according to the ketamine applications. The pro-inflammatory nutritional parameters included in the DII score is total calories (kcal); carbohydrates (g); fat (g); protein (g); cholesterol (mg); total saturated fatty acids (g); iron (mg); and vitamin B12 (μg). The anti-inflammatory dietary parameters included in the DII score is: alcohol (g); caffeine (g); fiber (g); total monounsaturated and polyunsaturated fatty acids (g); n-3 and n-6 polyunsaturated fatty acids (g); niacin (mg); riboflavin (mg); thiamine (mg); vitamins A (retinol equivalents), B6 (mg), C (mg), D (μg), E (mg); β-carotene (μg); magnesium (mg); selenium (μg); zinc (mg); and folate (μg). Scores are centered at 0, with positive scores indicating a pro-inflammatory diet and negative scores indicating an anti-inflammatory diet. Continuous DII scores is standardized (mean = 0, standard deviation = 1) for better interpretation of results.

### Outcome measures

Primary and secondary outcomes and endpoints that correspond to the secondary objectives are listed according to the various assessment time points in Table 1. As a primary outcome, this work is expected to prove the benefits of ketamine in the treatment for suicidal ideation in patients with MDD or BD, as well as the durability of the antidepressant effect, and the transdiagnostic comparison of the effect of ketamine. The C-SSRS scale score over 6 months in relation to the initial score is used to assess this primary outcome.

**TABLE 1:** Assessment schedule.

Assessments	Eligibility	1	2	3	4	5	6	7	8	Follow up (1 month)	Follow up (2 months)	Follow up (3 months)	Follow up (4 months)	Follow up (5 months)	Follow up (6 months)
Informed consent	X (Re-affirm)	X													
General data information		X													
Clinical information		X													
Nutritional measures		X							X						
Inflammatory profile of the diet		X		X		X		X							
CTQ		X													
Bloods (IL-6, IL-10, IL-1β, TNFα, SIRT3, suPAR, us-CRP and leptin)		X							X						
C-SSRS		X	X	X	X	X	X	X	X	X	X	X	X	X	X
MADRS	X	X	X	X	X	X	X	X	X	X	X	X	X	X	X
BPRS		X	X	X	X	X	X	X	X	X	X	X	X	X	X
YMRS	X	X	X	X	X	X	X	X	X	X	X	X	X	X	X
HAMD		X	X	X	X	X	X	X	X	X	X	X	X	X	X
FAST		X	X	X	X	X	X	X	X	X	X	X	X	X	X

As a secondary outcome, we are verifying the occurrence of changes before, during and after treatment on psychiatric scales (e.g., BPRS, MADRS, HAMD, YMRS, FAST) and serum concentration of IL-6, IL-10, IL-1β, TNFα, suPAR, us-CRP, and leptin as well as gene expression and immunocontent of SIRT3. In addition, the presence of MS is evaluated as a potential moderator of treatment response together with the predictors mentioned above.

The standard tools BPRS, MADRS, and HAM-D are used for comparison with other ketamine literature available in psychiatry (23, 24, 58, 59). Clinical response is defined as MADRS score reduction of ≥ 50% from baseline and remission as MADRS score ≤ 9 (22), and relapse is defined as MADRS ≥ 16 after an initial remission. The time points for measurements of the scales used were chosen to verify the initial and maximum response time (during treatment) and the duration of response (up to 6 months).

### Assessment integrity

Under the guidance of PBA, LNC and MKS (staff psychiatrists), the APA researcher participated in training to perform psychiatric assessments. APA then provides on-site initiation and training for the rest of the research team members (nutritionist, undergraduate students, and other researchers).

Eligible participants undergoing ketamine treatment are monitored regularly in each treatment application. Upon completion of treatment, participants go through the follow-up phase, in which they are monitored monthly by telephone (months 1–6) (Table 1). Each participant receives a unique identification number. All study data is recorded on the study case report forms and entered by study researchers into REDCap, a sophisticated platform for collecting and managing research data protected by Secure Sockets Layer encryption. All source documents and the master list linking participant identification information and identification numbers are stored in a locked cabinet at HCPA. All information is accessible only to those directly involved in the study. There is no advance sharing of data beyond the group of investigators. Study records are maintained for 5 years after study completion in secure archival facilities per the National Council for Health and Medical Research and Good Clinical Practice guidelines.

### Data analysis

We will use the Shapiro-Wilk test to assess the normality of the variables. Clinical and demographic data with normal distribution will be assessed with parametric tests (e.g., *t*-test for independent samples) and those with non-asymmetric data will be analyzed with non-parametric tests (e.g., Mann-Whitney test). Categorical variables will be compared through the chi-square test or Fisher’s exact test, as appropriate.

For the analysis of the C-SSRS suicidal ideation severity scale, as it is an ordinal scale, we plan to use the Wilcoxon test to detect differences between the scores for each evaluation point throughout the study. In addition, the generalized estimation equations will be used to evaluate the durability of the antidepressant effect and tolerability of SC ketamine.

The association between the variables is evaluated by the Pearson correlation test or Spearman, as per the distribution pattern. The margin of error used is 5%.

### Trial duration

July 2021–December 2023.

### Ethics and dissemination

This study is supervised by the Research Ethics Committee of *the Hospital de Clínicas de Porto Alegre* (CEP-HCPA) and Research Ethics Committee of the *Hospital Moinhos de Vento* (CEP-HMV). The same is a collegiate instance, of a consultative, deliberative, and educational nature, whose objective is to assess the ethical and methodological aspects (through the issuance of an opinion) and to monitor research projects involving human beings, carried out or proposed by the institution. The instance is registered with OHRP/USA (Office for Human Research Protections): IORG0000588, CEP Registration (IRB – Institutional Review Board) with OHRP/USA (Office for Human Research Protections): IRB 00000921 and Federal wide Assurance (FWA), certificate of commitment that the Institution undertakes to follow the requirements established by the HHS (U.S. Department of Health and Human Services) Protection of Human Subjects: FWA00002409.

This study was approved by Research Ethics Committee the HCPA (CAAE: 33589320300005327) on the 18 June 2020 and Research Ethics Committee the HMV (CAAE: 33589320.3.2001.5330). This trial has been registered in U.S. National Library of Medicine–Clinical Trial Registry (Reference Number: NCT05249309), with recruitment commenced on the May 2021.

The results of this study will be submitted for publication in peer-reviewed journals and presented at relevant conferences.

## Discussion

To the best of our knowledge, this study is the first naturalistic investigation designed to verify the therapeutic effects of SC ketamine in reducing suicide risk in patients with mood disorders. Furthermore, it is the first study to assess the impact of ketamine on serum levels of SIRT3, IL-6, IL-10, TNFα, leptin, us-CRP, suPAR, metabolic parameters, and dietary inflammatory index.

This study includes a period of ketamine administration (treatment) and a 6-months follow-up to determine not only the acute effects of ketamine but also its impacts in the long term. This duration was chosen to obtain adequate data on effect and durability in the short, medium, and long term, maintaining the feasibility of the study. Therefore, this study may provide relevant information for a future definitive study exploring the safety, tolerability, and effects of ketamine for suicidal ideation and/or behavior.

A study carried out in Denmark, observed that recent psychiatric hospitalization was the factor most strongly associated with suicide (60). This finding reinforces the idea that severe mental disorders is one of the leading indicators of suicide risk. Later, in 2010, mental disorders and substance use disorders were found to be responsible for two-thirds of suicides. Considering the additional burden of mental and substance use disorders as a risk factor for suicide, increased mental and substance use disorders have risen from the fifth most common disease category in the global burden to the third most common disease category (61).

The risk of suicide increases more than twenty-fold in individuals with DDM and is even greater in subjects with comorbidity with other psychiatric disorders or medical conditions (62). Psychological autopsy data show that approximately half of the individuals who died by suicide were suffering from depression. Lee et al. (7) observed that, compared to anxiety disorders, the diagnosis of MDD was associated with an odds ratio about ten times higher.

The causes of suicidal behavior are multiple and complex. Although the presence of MDD is an important predisposing factor, the existence of this pathology alone is not enough to fully explain suicidal behavior, without the interaction with other factors, such as the presence of hopelessness, impulsiveness, and aggression, among others. Furthermore, clinical predictors of suicidal behavior are generally not robust, meaning they are not reproducible for different patient samples, since suicidal behavior results from a combination of individual risk factors (63).

A systematic review recently verified that ketamine and esketamine are promising treatments for MDD, given their efficacy and tolerability (18). The authors analyzed 12 articles (two randomized controlled trials, five case reports and five retrospective studies). SC ketamine was administered to unipolar and bipolar patients in single or multiple doses, weekly or twice a week; the dose ranged from 0.1 to 0.5 mg/kg. In all studies, SC Ketamine showed a rapid and robust antidepressant effect, with remission rates of 50 to 100% after single or multiple doses, with transient side effects.

Neurobiological studies with adult suicide patients have found reduced levels of serotonin metabolites in central nervous system (CNS) fluid. Deficiency of this and other neurotransmitters (such as norepinephrine) has been observed in cases of suicide since the deficiency of these neurotransmitters in critical places in the brain results in depressive states. Such a deficiency can occur due to insufficient production, excessive neurotransmitter reuptake in the synaptic cleft or failure of the receptor system (64).

Serotonin and norepinephrine are the most studied neurotransmitters when it comes to suicide. Studies have also described the association of a decrease in the level of 5-HT in the brain of the deceased who had a diagnosis of depression. In the case of those who died by suicide, there was a decrease in 5-hydroxyindoleacetic acid (5-HIAA), the main metabolite of 5-HT. Depressed individuals who committed suicide or serious attempts had reduced levels of 5-HT, when compared to patients with depression, but who did not commit suicide or serious attempts (65).

Sirtuin 3 (SIRT3) is the main NAD + deacetylase dependent mitochondria that acts as a regulator of mitochondrial protein function, being essential for maintaining mitochondrial integrity. Abe et al. (66) analyzed whether there were alterations in sirtuin messenger RNA (mRNA) expression in peripheral white blood cells of BD patients and also examined whether altered sirtuin mRNA expression is state or characteristic dependent in BD patients who were in a remissive state. As a result, they observed that mRNA sirtuin levels in BD patients significantly decreased in those who were in a depressed state, compared to healthy controls. Therefore, altered sirtuin expression is state-dependent and is associated with the pathogenesis or pathophysiology of bipolar depression. A study correlated SIRT3 with depression, using semiquantitative Western blotting methods, associating the pathogenesis of depression with the expression of SIRT3 (67).

The urokinase plasminogen activating receptor (uPAR) is part of the plasminogen activation system. It is also involved in cell adhesion and migration and is important for the recruitment of immune cells (68). The soluble form of the receptor, suPAR, results from cleavage and release of membrane-bound uPAR into the blood and reflects activation of the immune system. In most cases, serum levels of suPAR positively correlate with inflammatory proteins such as TNFα and C-reactive protein (CRP) (41). It was also observed that high levels of suPAR were associated with a higher probability of diagnosis of depression (42). Ventorp et al. (43) evaluated plasma levels of suPAR as a biomarker of low-grade inflammation in patients with DDM and in patients who had recently attempted suicide. It was observed that both depressed patients and those who attempted suicide increased plasma suPAR, which may in the future be a prognostic in relation to the outcome of treatment with the application of conventional antidepressants in conjunction with anti-inflammatory drugs.

Patients with MDD are at increased risk for the development of metabolic and cardiovascular diseases, being, on average, 1.58 times more likely to have MS compared to the general population (35). On a global scale, it is estimated that 31% of patients diagnosed with BD have MS (35). MS is an umbrella term for clinical and biochemical changes, which include central obesity (waist circumference or body mass index), dyslipidemia (elevated triacylglycerols and reduced high-density lipoprotein-HDL), hyperglycemia, and hypertension (55). The presence of metabolic syndrome components in BD, mainly excess adiposity, is associated with reduced neurocognition, in addition to an association with executive function deficits and global cognitive deterioration (69). Excess central adiposity, assessed by waist circumference, is an important clinical marker in MS, because adipose tissue is recognized as an endocrine tissue that secretes hormones involved in several biological responses, including inflammation (70).

Among the hormones secreted by adipose tissue, leptin appears to be involved with depressive disorders, including MDD and depression in BD (36–39, 71–73). Leptin was described in 1995 as a hormone responsible for the feeling of satiety, which acts mainly on the CNS and inhibits the action of orexigenic neurons and stimulates the action of anorectic neurons, in addition to increasing basal energy expenditure and being secreted in proportion to the adipose tissue stock (74).

In addition to the metabolic aspects involved in mood disorders and suicide, nutritional aspects and diet quality have recently come to be considered in mental disorders (75), mainly as process-stimulating inflammatory agents. In a meta-analysis of prospective cohort studies, individuals grouped in extracts with better quality dietary patterns had a lower odds ratio for incidence of depression or depressive symptoms compared to individuals in extracts with lower quality dietary patterns (76). In the same study, those individuals who were in the lowest quintiles of the Dietary Inflammatory Index (DII) also had a lower odds ratio of incidence of depression and depressive symptoms (76), suggesting that nutritional factors may influence the development of mood disorders *via* stimulation of the inflammatory process. The dietary inflammatory index (IBD) is a global measure of the inflammatory potential of the foods consumed, considering macronutrients and micronutrients and their association with inflammatory markers such as IL-1b, IL-4, IL-6, IL-10, TNF –α, and CRP (57). In the case of MDD and suicidal ideation, the positive index of IBD, indicating a pro-inflammatory eating pattern, was associated with suicidal ideation and MDD in a cross-sectional study with a representative sample of the American adult population (56). In this sense, a systematic review of cross-sectional studies showed a positive association between a dietary pattern consisting of a high intake of red meat and derivatives and a low intake of fruits and vegetables with the concentration of CRP, IL-6 and IL-18 (77), all inflammatory markers involved in mood disorders. However, the clinical potential of IBD as a reference for the inflammatory profile of the diet still needs to be better characterized in patients with MDD and using ketamine.

The main limitation of this study is its inability to report the definitive efficacy of ketamine, since a multicenter, randomized, controlled clinical trial is required for assess these outcome. Secondly, severely depressed or psychotic patients who cannot consent will be excluded. Thirdly, patients remained on antidepressant medications. Therefore, we cannot rule out the possibility that the improvements in suicidal ideation and depressive symptoms are due to the intensifying effects of ketamine and not just ketamine which would require a RCT. However, this problem can be partially solved based on the well-known antidepressant effect of rapid rise and fall of ketamine compared to gradual changes of antidepressants that take weeks to months. Fourth: have not evaluate criteria for Treatment-Resistant Depression (TRD) and, given the naturalistic design, we cannot exclude that part of the sample could be TRD. Lastly, the lack of blinding and placebo group to analyze the study primary outcomes (clinical outcomes), as the control group will be used only to analyze the biochemical markers.

The use of standard psychiatry research instruments (e.g., MADRS, YMRS, FAST, HAM-D, and BPRS) allows direct comparison of this study with other psychiatric studies. In particular, using YMRS and BPRS allow for a better characterization of the side effect of ketamine confusion on various psychotomimetic and dissociative symptoms as well as manic symptoms. The C-SSRS is widely used in research that evaluates other drugs for suicidal ideation and has high impact power (43–45). The Centers for Disease Control and Prevention (CDC) and the FDA also recommend using the C-SSRS as a reference predictive measure in the analysis of suicide-related issues.

This protocol provides important information for a future definitive study exploring the safety, tolerability, and effects of ketamine for suicidal ideation and/or behavior in addition to investigating the specific molecular mechanism behind the immunomodulatory effects of ketamine.

## Ethics statement

This study was approved by Research Ethics Committee the HCPA (CAAE: 33589320300005327) and Research Ethics Committee the HMV (CAAE: 33589320.3.2001.5330). Participants consent by signing the consent form.

## Author contributions

MK-S, PB-D-A, and AA prepared the body of the protocol. AA, MK-S, PB-D-A, KC, and JVP prepared all information regarding sample size and statistical analysis. MK-S, JVP, PB-D-A, and LC contributed to the preparation of the entire body of the protocol. AA, JG, VC, and KC prepared study flow and assessment description, table, diagram, references, and formatted the manuscript. All authors read and approved the final manuscript.

## Appendix

**APPENDIX 1:** Patient assessment form.

General patient data sheet
Name, number, and initials:
Have you been diagnosed with depression by a psychiatrist?
Have you had depressive symptoms for the past 2 weeks most of the time?
Are you taking any psychotropic medication (antidepressants, antipsychotics, tranquilizers or mood stabilizers)? If so, which drugs and what doses?
-
-
–
Have you been taking the same medications at the same doses for more than 4 weeks in a row daily?
Have you started any psychotherapy (psychological follow-up) recently? If yes, how long ago?
Do you have any known medical illnesses? If yes, what diseases?
